# Supplementary material for: Quantitative metaproteomics of medieval dental calculus reveals individual oral health status
Source: Nat Commun. 2018 Nov 20;9:4744. doi: 10.1038/s41467-018-07148-3 (PMC6246597; doi:10.1038/s41467-018-07148-3)
Supplement: Supplementary file 1 — Supplementary Information [file 41467_2018_7148_MOESM1_ESM.pdf]

Supplementary Information

Quantitative metaproteomics of medieval dental calculus reveals individual oral health status

Jersie-Christensen, et al.

Supplementary Note 1. Bioarchaeological sampling strategy

In order to look at the diversity in microbiome, as well as disease biomarkers from dental calculus, we were interested in individuals most likely to have osseous changes and destruction of the alveolar bone related to periodontitis. Older males were selected based on the understanding that males often have a more aggressive inflammatory immune response compared to females[1,2,3]. Males over 45 with a maxilla, mandible, teeth, evidence of periodontal disease, and sufficient dental calculus for sampling, were the initial criteria for inclusion. However, this yielded only 12 samples. By adding younger males, it was possible to collect 22 dental calculus samples from 21 individuals. Samples were taken from the largest deposits available, but sometimes samples had to be aggregated, as a single tooth did not have enough material available (see Supplementary Table 2).

Individuals were scored for every tooth position based on different criteria according to the references cited in the main text. Scores of antemortem tooth loss (AMTL = score 7, n = 50) were conservative, and only assigned when there was extensive or complete remodelling of the alveolar bone. In some instances teeth must have been retained largely due to the soft tissues of the periodontium, because remodelling of the alveolus was so substantial that teeth could not be placed back into the remaining 'sockets.' Teeth missing otherwise were considered lost postmortem (PMTL = score 6, n = 71), or not present (NP = score 8, n = 17). Teeth assigned NP were considered impacted or congenitally absent. Scores of 9 were allotted in the case of missing data, such as where scores were not possible due to destruction or loss of the bone or tooth, the presence of glue, or other obfuscating factors (see Supplement 1D).

The cemetery of Tjærby was chosen as the material is well-curated, and because it represents a complete medieval assemblage. This allows for larger numbers of remains (in this case older males), to be analysed from an intra-site perspective. Being medieval, preservation of biomolecules should theoretically be better than earlier periods, but the material also predates major changes to diet and lifestyle that occur in the post medieval period. Social stratification in a rural parish cemetery should be limited. Therefore, through this relative homogeneity, we can assess closely as possible to an 'average medieval person's' microbiome.

Supplementary Note 2. Brief osteological notes on each individuals

A795 (#1) (Male 36-45): Most scores for periodontal disease are 2 with a couple of 3s, the LL6 is almost completely destroyed by caries, and there are three small carious lesions. Much of the anterior teeth are lost postmortem. Pathology score +--

A970 (#19) (Male 36-45): Mostly periodontal scores of 2, but one score of 4. A few gross and smaller carious lesions, abscesses (UL8 drains in the maxillary sinus), and antemortem tooth loss. The anterior alveolus is damaged and some of the incisors are lost postmortem. Pathology score +-+

A983 (#18) (Male 45+): Removed from bioarchaeological analysis because the teeth were too worn for adequate identification and there was extensive antemortem tooth loss. Sampled tooth (buccal UR7) was covered down along the roots. Pathology score: N/A

A1235 (#20) (Male 45+): Nine teeth lost antemortem, so periodontal disease may have been advanced. No evidence of caries, and no abscesses, but one periapical cyst The dental calculus sampled from UR8 was green along gingival margin. Pathology score -++.

A1294 (#9) (Male 45+): The individual has six gross carious lesions and four teeth lost antemortem, dental calculus is mostly absent in mandible. Alveolus is damaged in places. Pathology score ++-

A1408 (#2 & #10) (Male 45+): A high degree of antemortem tooth loss (6 maxilla, 3 mandible), the mandible has high scores for dental calculus and periodontal disease is advanced and generalized (mostly scores of 3, but no 4). There are two samples from this individual, one is a 'normal' sample from the maxillary right canine. The other, is a sample of 'verrucous' dental calculus that may relate to orofacial pathology. Pathology score -++

A1416 (#3) (Male 45+): Wear is generally high, and there is pulpal breach on of a number of the molars. Periodontal disease is generally 2, but two scores of 4 (not associated with dental calculus). One large carious lesion, one periapical granuloma, and three large abscesses. Pathology score +-+

A1442 (#11) (Male 45+): Dental calculus and periodontal disease are more advanced in the mandible (one score of 4). No dental caries, two abscesses, a cyst, and the left maxillary molars are lost antemortem. Wear quite advanced. Pathology score -++

A1453 (#21) (Male 45+): A lot of oral pathology, but periodontal disease seems to be more localized with three 4s, but also scores of 1. The alveolar bone crest is especially porous in places. Wear is very heavy, and hypercementosis is present. A few large carious lesions, and abundant periapical lesions (abscesses x 6, a granuloma, and a cyst): six teeth lost antemortem. Pathology +++

A1623 (#12) (Male 36-45): Much heavier wear in the maxilla; more advanced periodontal disease (seven 4 scores) than the mandible (two 4 scores), but the mandible has more dental calculus. The right side has two small and one large carious lesion, and two abscesses. Pathology score +-+

A1635 (#13) (Male 36-45): Generalized and mild periodontal disease, dental calculus is minor, especially in maxilla, no caries or periapical lesions. Pathology score ---

A1637 (#4) (Male 45+): Dental calculus and periodontal disease is more severe in the mandible. There are no dental caries, but three abscesses (one drains in the maxillary sinus, another into the mandibular corpus). Pathology score --+

A1664 (#23) (Male 36-45): Quite heavy wear with pulpal breach of the first molars. A couple of small caries, but large abscesses, and three periapical cysts (including a massive one). The left maxilla is missing (postmortem). Florid periosteal reactionary new bone on the left tibia and fibula. Pathology score ---

A1671 (#5) (Male 36-45): Relatively low dental calculus for the amount of periodontal disease (two 4 scores), dental caries are absent, but there is one abscess and two teeth lost antemortem. Pathology score --+

A1673 (#6) (Male 36-45): Most teeth are glued in, so hard to measure, but most positions score 3 for PD in the mandible, and calculus is heavy. Some small carious lesions, and two abscesses. Pathology score --+

A1764 (#14) (Male 26-35): Youngest individual, with mild to moderate periodontal disease present throughout, also six carious lesions are in four mandibular molars, one is abscessed. Pathology score +--

A1866 (#22) (Male 45+): Periodontal disease is generalized with mostly scores of 2, and seven scores of 3, and two teeth have 4 scores for calculus. Six teeth lost antemortem (most in mandible), and there is one abscess. Pathology score -+-

A1893 (#15) (Male 45+): Mild periodontal disease present at most locations, but no teeth lost antemortem and only one moderate cervical carious lesion. Pathology score ---

A1898 (#16) (Male 36-45): ‘Healthiest’ individual, with some scores of 2, low dental calculus except on mandibular incisors, no dental caries and no periapical lesions, all teeth present. Pathology score ---

A1899 (#7) (Male 45+): The right side is overwhelming affected by oral pathology, the alveolar bone crest has been damaged extensively in the maxillary cheek teeth by abscesses and periodontal disease. There are two teeth mostly destroyed by caries. Hypercementosis noted on some teeth. Pathology score +-+

A1968 (#24) (Male 45+): One tooth lost antemortem, PD is mostly absent from maxilla, but present and mild in mandible due to larger calculus build-up. One cyst and one small carious lesion. Pathology score ---

Supplementary Note 3. Document from Danish ethics committee

The regional science ethics committee in the Copenhagen area was contacted and the chief consultant assessed that the study did not require notification to the ethics committee, see letter next pages (in Danish). English translation after the letter.

Region Hovedstaden

Center for Sundhed

De Videnskabsetiske Komiteer

Regionsgården
Kongens Vænge 2
3400 Hillerød

Opgang B+D
Telefon 3866 6395
Direkte +4538666320
Mail vek@regionh.dk

PhD student
Rosa Rakownikow Jersie-Christensen
University of Copenhagen
NNF CPR Rroteomics
Blegdamsvej 3B
2200 Copenhagen N

Protokol nr.:

Journal nr.: H-17010124
Ref.: Lone Gundelach

Dato: 6. april 2017

Undersøgelse af sammensætningen af bakterielle proteiner samt evt. humane proteiner i plak fra museum-prøver.

I mail af 28. marts 2017 har du spurgt, om en undersøgelse, du lavede i forbindelse med ovennævnte projekt, skulle have været anmeldt til en videnskabsetisk komite.

Du oplyser, at du indsamlede plak og tandsten fra 7 raske frivillige med det formål at teste, hvilke proteiner, I kunne identificere ved hjælp af massespektrometri.

Jeg har vurderet, at der ikke var tale om et sundhedsvidenskabeligt forskningsprojekt som dette er defineret i komitélovens § 2,[1], men at der var tale om et metodestudie.

Projektet var derfor ikke anmeldelsespligtigt, jf. komitélovens § 1, stk. 4 og kunne iværksættes uden tilladelse fra De Videnskabsetiske Komiteer for Region Hovedstaden.

Der ligger således ikke i afvisningen af at bedømme projektet nogen etisk stillingtagen eller negativ vurdering af dets indhold.

<u>Klagevejledning:</u>

Komitéens afgørelse kan, jf. komitélovens § 26, stk. 1, indbringes for National Videnskabsetisk Komité, senest 30 dage efter afgørelsen er modtaget. National Videnskabsetisk Komité kan, af hensyn til sikring af forsøgspersonernes rettigheder, behandle elementer af projektet, som ikke er omfattet af selve klagen.

Klagen skal indbringes elektronisk og ved brug af digital signatur og kryptering, hvis protokollen indeholder fortrolige oplysninger.

Dette kan ske på adressen: dketik@dketik.dk

[1] Lov nr. 593 af 14. juni 2011 om videnskabsetisk behandling af sundhedsvidenskabelige forskningsprojekter med senere ændring.

Klagen skal begrundes og være vedlagt kopi af Den Regionale Videnskabsetiske Komités afgørelse samt de sagsakter, som Den Regionale Videnskabsetiske Komité har truffet afgørelse på grundlag af.

NB: Der må ikke foretages ændringer i dokumenterne, som har været til behandling i komiteen, da sagens ellers vil blive sendt retur til komiteen.

Med venlig hilsen

Lone Gundelach
Chefkonsulent, cand.jur.
Lone.gundelach@regionh.dk

English translation:

Examination of the composition of bacterial proteins as well as possibly human proteins, in plaque from museum samples.

By mail of 28 March 2017 you have asked if a study you made in connection with the above project should have been reported to a scientific committee.

You inform that you collected plaque and calculus from 7 healthy volunteers for the purpose of testing which proteins you could identify using mass spectrometry.

I have considered that it was not a health science research project as defined in Section 2* of the Committee Act, but that it was a method study.

The project was therefore not required to report according to Section 1, Subsection 4 of the Committee Act. and could be implemented without permission from the Scientific Ethics Committees for the Capital Region.

Thus, there is no judgement of the project in any ethical aspects or negative assessment of its content.

Complaints:

The Committee's decision can, according to Section 26, Subsection 1 of the Committee Act. be filed to the National Science Ethics Committee, no later than 30 days after the decision has been received. The National Science Ethics Committee may, for the purpose of ensuring the rights of subjects, treat elements of the project that are not covered by the complaint itself.

The complaint must be filed electronically and using digital signature and encryption if the protocol contains confidential information.

This can be done at: dketik@dketik.dk

The complaint must be justfied and hold a copy of the decision of the Regional Scientific Committee and the files used by the Regional Scientific Committee as the basis of their decision.

NB: No changes may be made to the documents that have been submitted to the committee, as the case will otherwise be returned to the committee.

Kind Regards

Lone Gundelach

Chief consultant, cand.jur

* Act No. 593 of 14 June 2011 on scientific ethical treatment of health science research projects with later change.

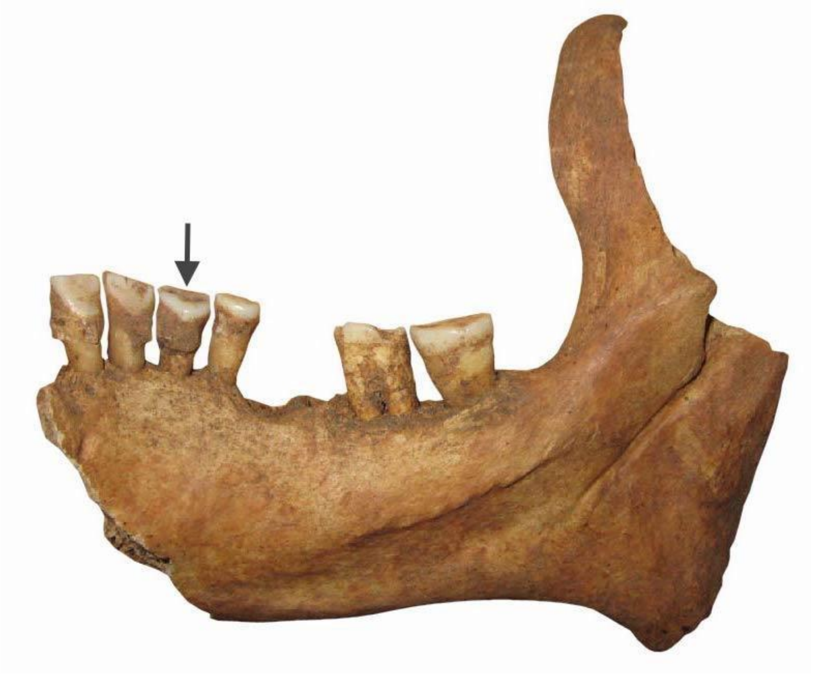

Supplementary Figure 1. Sampling location of dental calculus from Tjærby #22 (A1866).

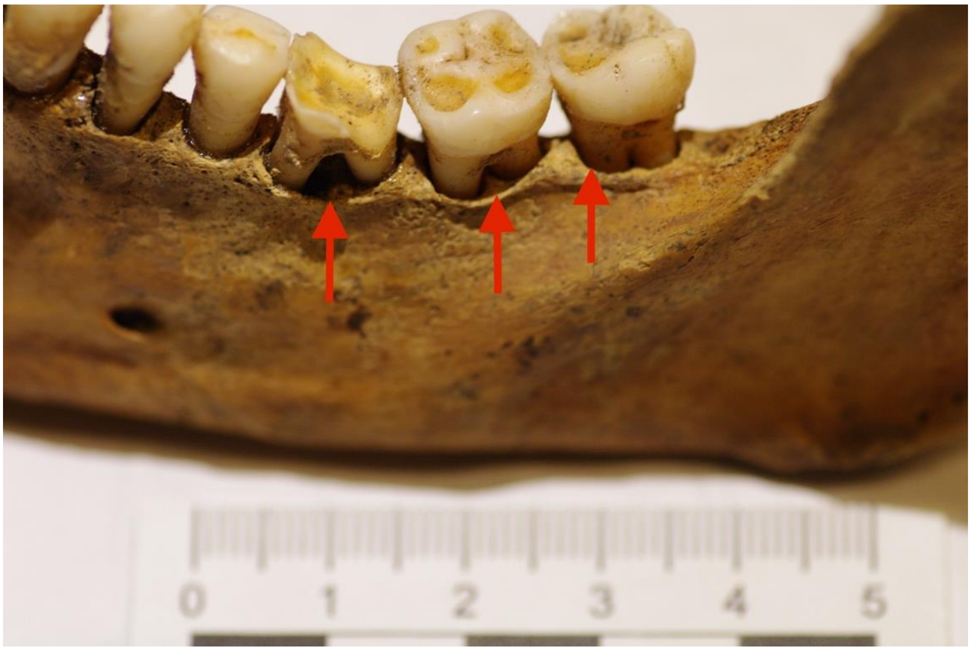

Supplementary Figure 2. The photograph of #6 (A1673) showing periodontal troughs (red arrows) along the buccal edge of the left molars (note also glue in the socket of LL5).

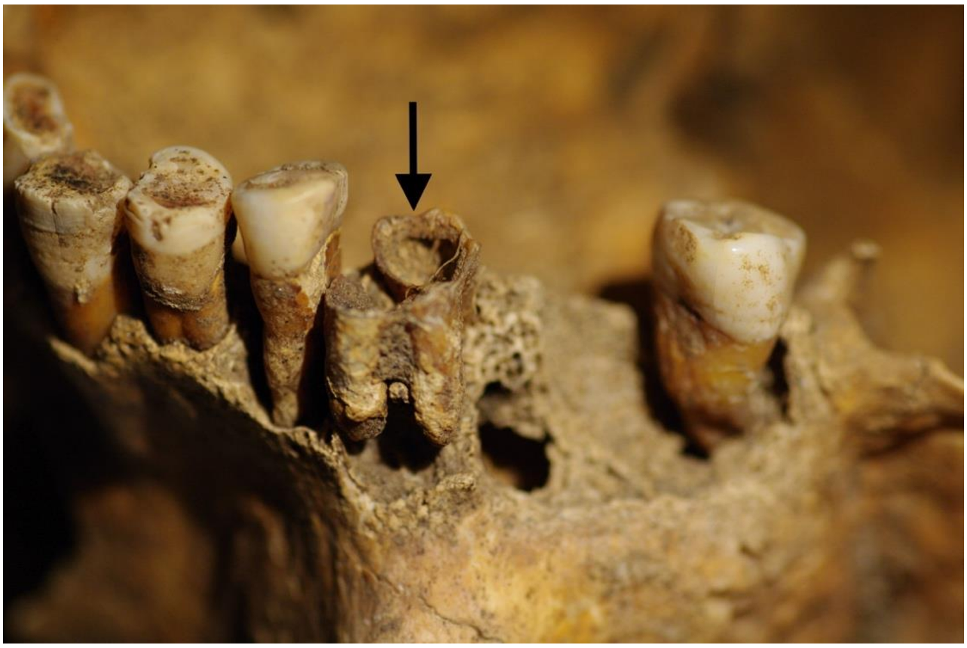

Supplementary Figure 3. A large carious lesion (black arrow) that has destroyed the crown of the upper right first molar with associated abscess formation in individual #7 (A1899).

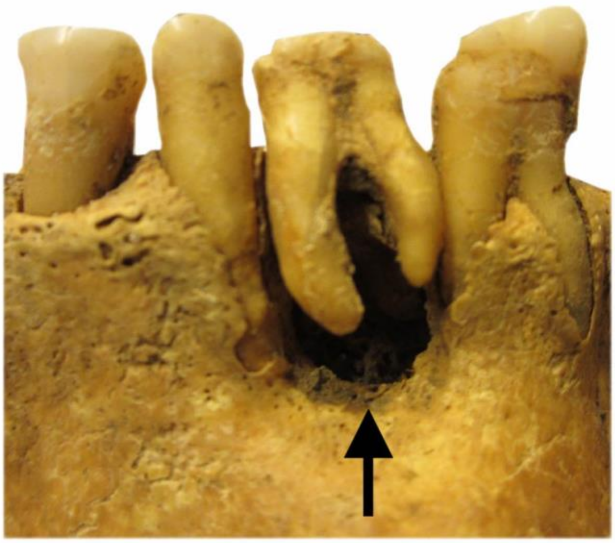

Supplementary Figure 4. Large abscess (black arrow) corresponding to the upper right first molar of #11 (A1442).

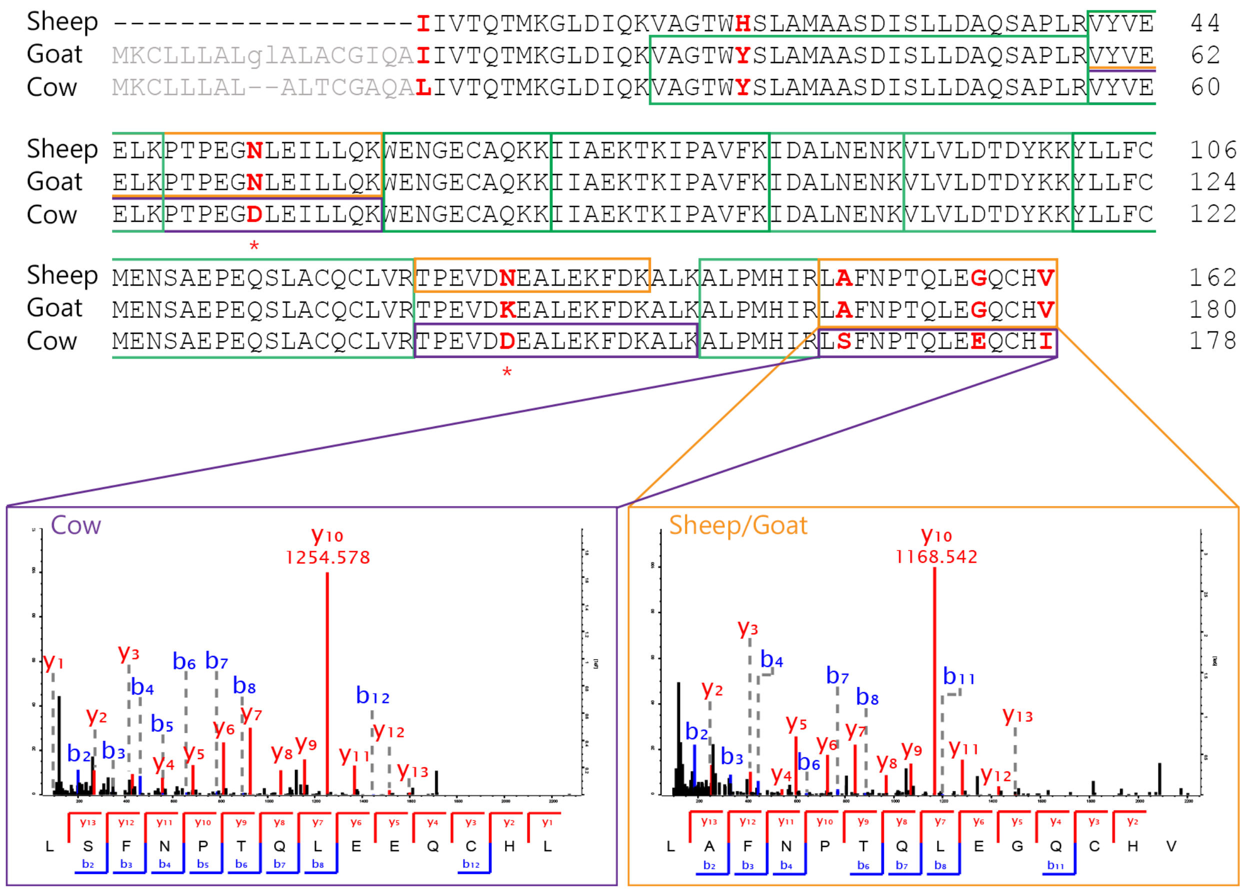

Supplementary Figure 5. Amino acid sequence of beta lactoglobulin from sheep, goat and cow.

All boxed sequences are identified in our MS analysis. Green boxes indicate common peptides found, orange unique to sheep/goat and purple unique to cow. Only the C-terminal peptides are used for species identification, since the others only differ between N and D, which could also correspond to a deamidation.

a

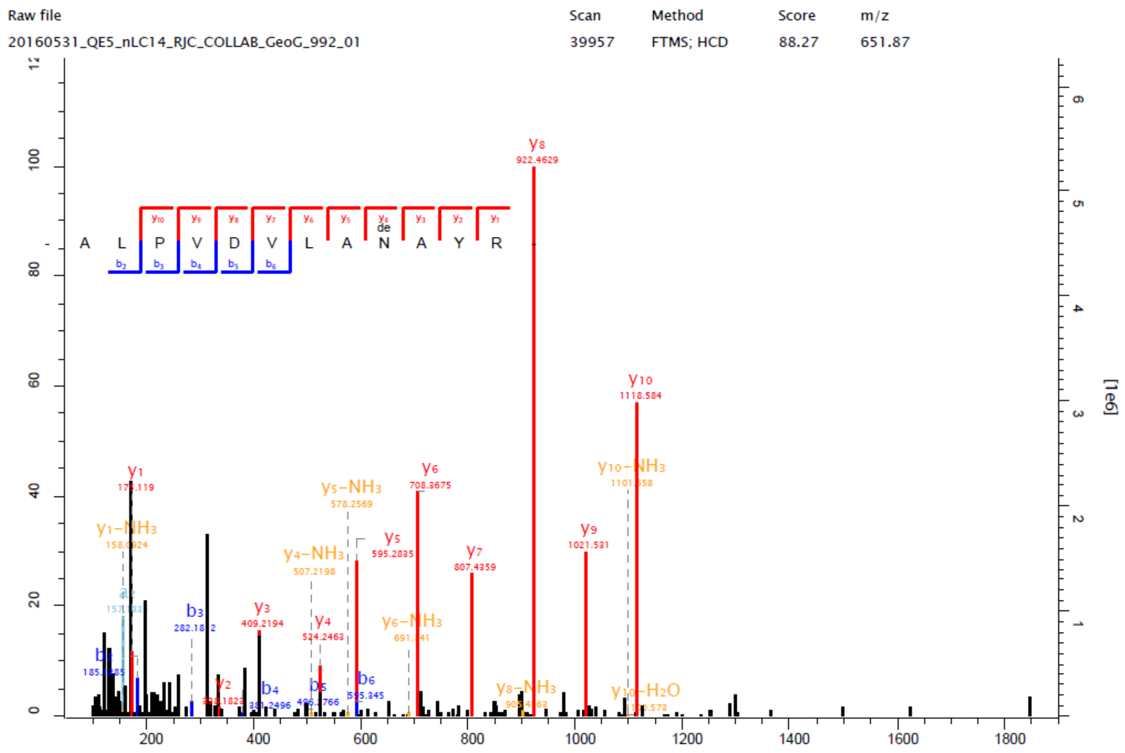

b

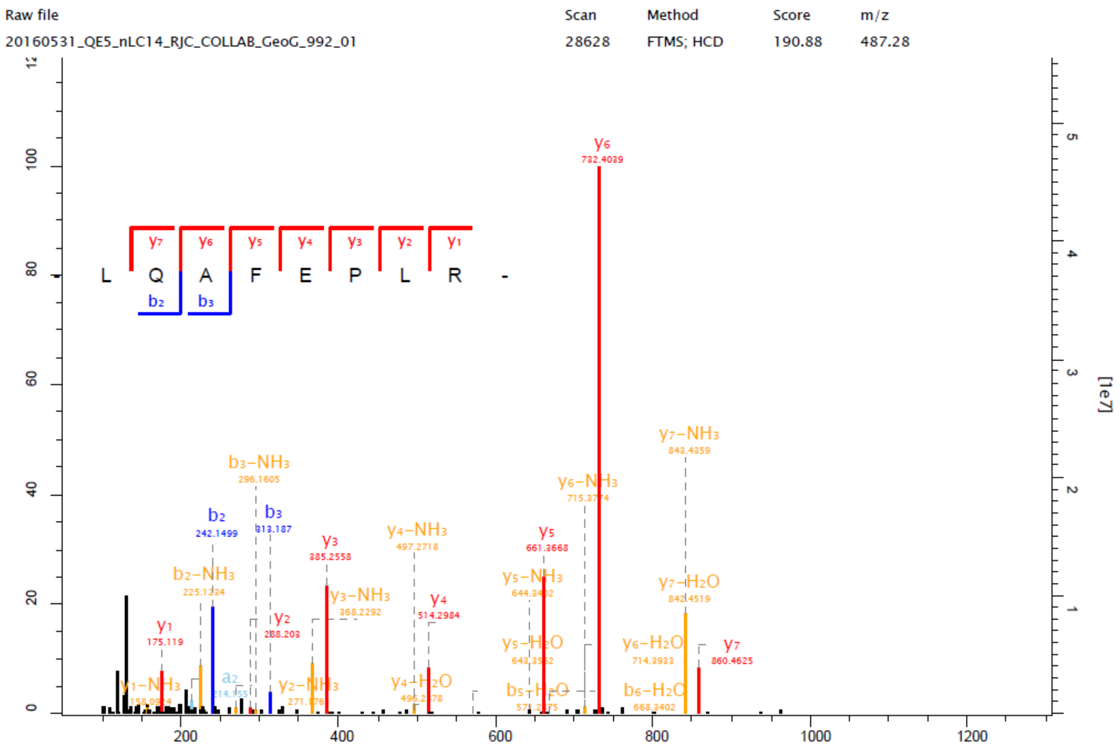

Supplementary Figure 6. MS2 spectra of two peptides identified as oat. These two peptide sequences can be mapped to seed storage globulin of *Avena sativa* (Oat).

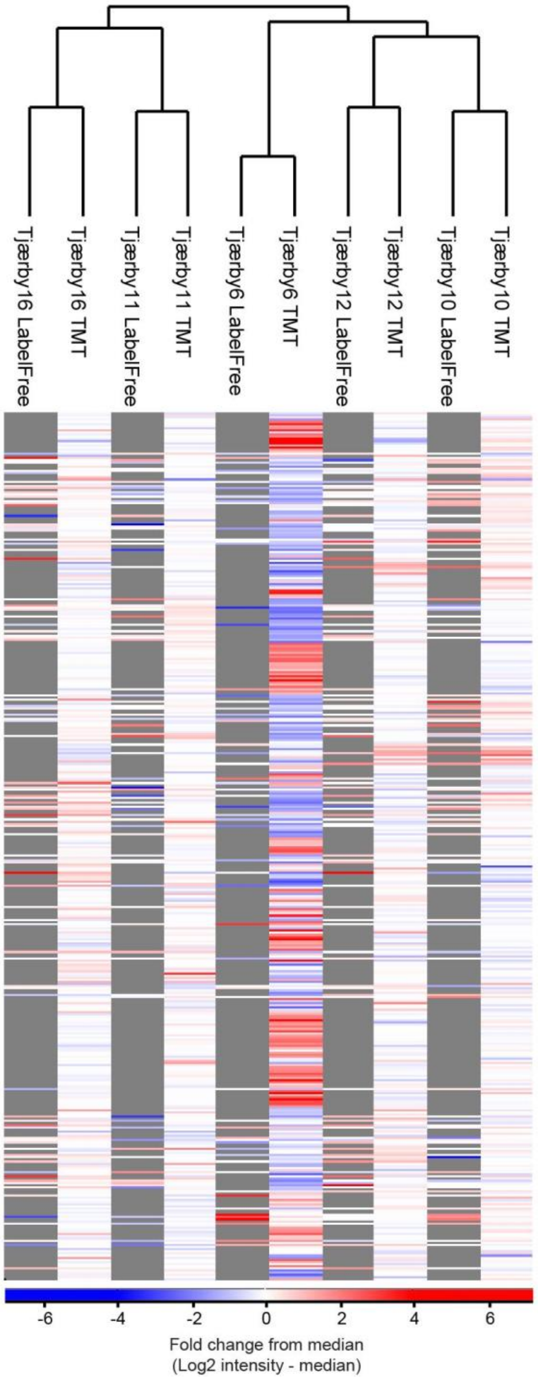

Supplementary Figure 7. Hierarchical clustering of TMT labeled samples together with corresponding unlabeled sample. Tjærby 6 shows a different profile from the other samples because this is the only sample belonging to G2 and it was one of the more protein rich samples. The correlation between the labeled and unlabeled sample is good despite the missing values.

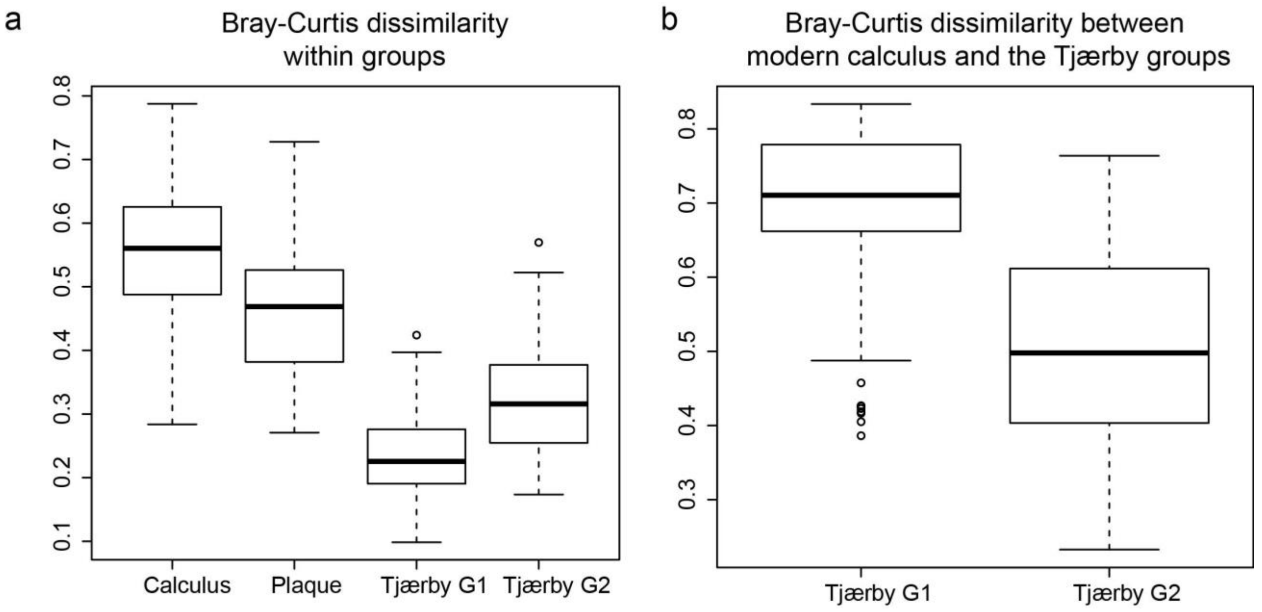

Supplementary Figure 8. Box plot of distribution of Bray-Curtis dissimilarity, with the value of 0 being similar and 1 being dissimilar. The box represents the second and third quartile and the line the median. Whiskers illustrates minimum and maximum values. a) Distribution of Bray-Curtis dissimilarity within the groups. For calculus and plaque representing 21 comparisons, G1 120 comparisons and G2 5 comparisons. The Tjærby samples shows less diversity (more intra-homogeneity) than both the modern sample groups. b) Distribution of Bray-Curtis dissimilarity between modern calculus and the two subgroups of Tjærby samples showing Tjærby G2 to be more similar to modern calculus. G1 represents 112 comparisons and G2 42 comparisons.

Supplementary Table 1. Pathology scores

Sample/Accession number for each individual	Gross caries 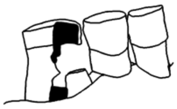	Teeth lost antemortem 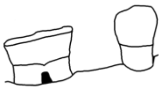	Presence of periodontal troughs 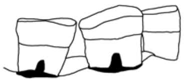	Total Score
#20 A1235	-	+	+	- + +
#2 / 10 A1408	-	+	+	- + +
#11 A1442	-	+	+	- + +
#9 A1294	+	+	-	+ + -
#12 A1623	+	-	+	+ - +
#3 A1416	+	-	+	+ - +
#7 A1899	+	-	+	+ - +
#19 A970	+	-	+	+ - +
#14 A1764	+	-	-	+ - -
#1 A795	+	-	-	+ - -
#4 A1637	-	-	+	- - +
#24 A1968	-	-	-	- - -
#15 A1893	-	-	-	- - -
#13 A1635	-	-	-	- - -
#16 A1898	-	-	-	- - -

**#18* A983**	**N/A**	**N/A**	**N/A**	**N/A**
**#21 A1453**	+	+	+	+ + +
**#5 A1671**	-	-	+	- - +
**#6 A1673**	-	-	+	- - +
**#22 A1866**	-	+	-	- + -
**#23 A1664**	-	-	-	- - -

Pathology Scores based on the presence of gross caries, >2 teeth lost antemortem, and the presence of periodontal troughs, from unhealthiest with three positive scores to healthiest with three negative scores (see also Supplementary Table 2). The bold line separates Group 1 from Group 2.

*Removed due to heavy wear and damage to teeth and surrounding bone.

Supplementary Table 2. Oral disease scores for each sample/individual.

Sample/ Accession number for each individual	Sex / Age	Dating (CE)[1]	Path-ology Score	Brad-ford Score of 3 #	Brad-ford Score of 4 #	Total Perio Obser-vations #	AMTL[2] #	Caries – gross #	Caries – minor #	Absce sses #	Cyst/ Granu-loma #	Dental Calculus[3]	Wear[3]
#1 A795	M 36-45	1100-1350	+--	2	0	15	0	1	3	0	0	L	H
#2 A1408	M 45+	1400-1450	--+	12	0	15	9	0	0	2	0	H	H
#3 A1416	M 45+	1100-1350	+-+	2	2	24	1	1	0	3	1	M	H
#4 A1637	M 45+	1100-1350	--+	11	0	18	2	0	0	3	0	H	M
#5 A1671	M 36-45	1100-1350	--+	2	2	14	2	0	0	1	0	M	H
#6 A1673	M 36-45	1250-1400	--+	21	0	29	1	0	2	2	0	H	M
#7 A1899	M 45+	1250-1400	+-+	8	1	18	0	2	2	7	0	M	H
#8 BLANK													
#9 A1294	M 45+	1250-1400	++-	1	0	6	4	6	2	1	0	L	H
#10 A1408	M 45+	1400-1450	-++	12	0	15	9	0	0	2	0	H	H

#11 A1442	M 45+	1100-1350	-++	2	1	11	3	0	0	2	1	M	VH
#12 A1623	M 36-45	1100-1350	+-+	9	7	23	1	1	2	3	0	M	H
#13 A1635	M 36-45	1250-1400	---	3	0	21	0	0	0	0	0	L	M
#14 A1764	M 26-35	1250-1400	+--	5	0	19	2	3	1	1	0	H	H
#15 A1893	M 45+	1250-1400	---	0	0	23	0	0	1	0	0	M	M
#16 A1898	M 36-45	1250-1400	---	0	0	30	0	0	0	0	0	H	M
#17 BLANK													
#18 A983*	M 45+	1100-1350	N/A	N/A	N/A	N/A	14	N/A	N/A	5	0	N/A	VH
#19 A970	M 36-45	1100-1350	+-+	2	1	16	2	2	4	2	0	L	M
#20 A1235	M 45+	1100-1350	-++	2	1	11	9	0	0	0	1	L	M
#21 A1453	M 45+	1100-1350	+++	7	3	21	6	2	1	6	2	M	VH
#22 A1866	M 45+	1100-1350	-+-	7	0	22	6	0	0	1	0	H	M
#23 A1664	M 36-45	1250-1400	---	4	0	18	1	0	3	2	3	M	VH
#24 A1968	M 45+	1100-1350	---	2	0	21	1	0	1	0	1	H	H
#25 BLANK													

[1]CE = common era

[2]AMTL = antemortem tooth loss

[3]H = heavy, M = moderate, L = light

*Removed from bioarchaeological analysis due amount of antemortem tooth loss and advanced age.

Supplementary Table 3. Dental calculus sample notes.

Sample / Accession number for each individual	Sample Location	Teeth Sampled	Sample Weight
#1 A795	Crown / root	Buccal UR8 and labial root LR6	18.2 mg
#2 A1408	Crown	Distal and lingual 'verrucous' calculus extending onto the roots from the LL8	145.4 mg
#3 A1416	Crown / root	Distal UL8, buccal roots UR6, buccal roots UL7	15.5 mg
#4 A1637	Crown	Lingual and buccal LL7 and LL1	42.5 mg
#5 A1671	Crown	Lingual LL1	19.2 mg
#6 A1673	Crown	Lingual LR1	31.0 mg
#7 A1899	Crown	Lingual LL5, LL8, and LR2	37.7 mg
#8 BLANK			
#9 A1294	Crown / root	Distal supragingival UL8 and buccal roots UR6	23.0 mg
#10 A1408	Crown	'Normal' sample, labial UR3.	25.9 mg
#11 A1442	Crown	Lingual and buccal LL7	34.2 mg
#12 A1623	Crown / root	Buccal CEJ LR2, LR3, LR4, and LR5	15.6 mg
#13 A1635	Crown	Buccal and lingual UR7 and Lingual UR6	32.9 mg
#14 A1764	Crown	Labial UR2 and UR3	39.0 mg
#15 A1893	Crown	Buccal UL7 and UR4	25.9 mg
#16 A1898	Crown	Buccal and lingual UL6, UR1, and UR2	30.1 mg
#17 BLANK			
#18 A983*	Root	UR7	127.0 mg
#19 A970	Crown	Labial LR4, LR6, and distal LR8	26.1 mg
#20 A1235	Crown	Distal and mesial UR8	23.2 mg
#21 A1453	Crown / root	LR3 (lingual root, M/D/B crown) and LL2 lingual root	31.2 mg
#22 A1866	Crown	Buccal and lingual LR4	45.9 mg
#23 A1664	Crown	Buccal LR3 and LR1	35.8 mg
#24 A1968	Root	Buccal root LL6	34.0 mg
#25 BLANK			

Supplementary References

1. Shiau, H. J. & Reynolds, M. A. Sex differences in destructive periodontal disease: exploring the biologic basis. *J. Periodontol.* **81,** 1505–1517 (2010).

2. Krustrup, U. & Petersen, P. E. Periodontal conditions in 35–44 and 65–74-year-old adults in Denmark. *Acta Odontol. Scand.* **64,** 65–73 (2006).

3. Scannapieco, F. A. & Cantos, A. Oral inflammation and infection, and chronic medical diseases: implications for the elderly. *Periodontol. 2000* **72,** 153–175 (2016).
