# Supplementary material for: Quantitative metaproteomics of medieval dental calculus reveals individual oral health status
Source: Nat Commun. 2018 Nov 20;9:4744. doi: 10.1038/s41467-018-07148-3 (PMC6246597; doi:10.1038/s41467-018-07148-3)
Supplement: Supplementary file 3 — Description of Additional Supplementary Files [file 41467_2018_7148_MOESM3_ESM.pdf]

Description of Additional Supplementary Files

File Name: Supplementary Data 1
Description: All bacterial genera identified

File Name: Supplementary Data 2
Description: All bacterial species identified
